# The globin gene family of the cephalochordate amphioxus: implications for chordate globin evolution

**DOI:** 10.1186/1471-2148-10-370

**Published:** 2010-11-30

**Authors:** Bettina Ebner, Georgia Panopoulou, Serge N Vinogradov, Laurent Kiger, Michael C Marden, Thorsten Burmester, Thomas Hankeln

**Affiliations:** 1 Institute of Molecular Genetics, Johannes Gutenberg-University, D-55099 Mainz, Germany; 2 Max-Planck Institute of Molecular Genetics, D-14195 Berlin, Germany; 3 Department of Biochemistry and Molecular Biology, Wayne State University School of Medicine, Detroit, Michigan 48201, USA; 4 INSERM, U473, F-94276 Le Kremlin Bicetre Cedex, France; 5 Biocenter Grindel and Zoological Museum, University of Hamburg, D-20146 Hamburg, Germany

## Abstract

**Background:**

The lancelet amphioxus (Cephalochordata) is a close relative of vertebrates and thus may enhance our understanding of vertebrate gene and genome evolution. In this context, the globins are one of the best studied models for gene family evolution. Previous biochemical studies have demonstrated the presence of an intracellular globin in notochord tissue and myotome of amphioxus, but the corresponding gene has not yet been identified. Genomic resources of *Branchiostoma floridae *now facilitate the identification, experimental confirmation and molecular evolutionary analysis of its globin gene repertoire.

**Results:**

We show that *B. floridae *harbors at least fifteen paralogous globin genes, all of which reveal evidence of gene expression. The protein sequences of twelve globins display the conserved characteristics of a functional globin fold. In phylogenetic analyses, the amphioxus globin BflGb4 forms a common clade with vertebrate neuroglobins, indicating the presence of this nerve globin in cephalochordates. Orthology is corroborated by conserved syntenic linkage of *BflGb4 *and flanking genes. The kinetics of ligand binding of recombinantly expressed BflGb4 reveals that this globin is hexacoordinated with a high oxygen association rate, thus strongly resembling vertebrate neuroglobin. In addition, possible amphioxus orthologs of the vertebrate globin X lineage and of the myoglobin/cytoglobin/hemoglobin lineage can be identified, including one gene as a candidate for being expressed in notochord tissue. Genomic analyses identify conserved synteny between amphioxus globin-containing regions and the vertebrate *β-globin *locus, possibly arguing against a late transpositional origin of the *β-globin *cluster in vertebrates. Some amphioxus globin gene structures exhibit minisatellite-like tandem duplications of intron-exon boundaries ("mirages"), which may serve to explain the creation of novel intron positions within the globin genes.

**Conclusions:**

The identification of putative orthologs of vertebrate globin variants in the *B. floridae *genome underlines the importance of cephalochordates for elucidating vertebrate genome evolution. The present study facilitates detailed functional studies of the amphioxus globins in order to trace conserved properties and specific adaptations of respiratory proteins at the base of chordate evolution.

## Background

Globins are heme-containing proteins that bind O_2 _and other gaseous ligands between the iron atom at the center of the porphyrin ring and a histidine residue of their polypeptide chain [1]. In addition to supporting aerobic metabolism of cells by providing O_2 _supply, globins fulfill a broad range of other functions, including O_2 _sensing, detoxification of harmful reactive oxygen species (ROS), the generation of bioactive gas molecules like NO and others [2]. Thus it is not surprising that the versatile globins are found from bacteria to fungi, protists, plants, and most animal groups [3].

The intensively studied hemoglobins (Hb) and myoglobins (Mb) are present in almost all vertebrate species, being responsible for O_2 _transport and storage, but also the production and elimination of NO [4,5]. Some years ago, the vertebrate globin gene family was expanded by the discovery of two additional globin types, neuroglobin and cytoglobin [6-8]. Neuroglobin (Ngb) is preferentially expressed in neurons and endocrine cells, and its expression patterns suggest an association with oxidative metabolism and the presence of mitochondria [9,10]. Cytoglobin (Cygb) is expressed in fibroblast-related cell types and distinct neuronal cell populations [11,12]. The exact physiological function(s) of both proteins are still uncertain, and several, partially contradictory hypotheses have been proposed, including functions in O_2 _supply, ROS detoxification, signal transduction and inhibition of apoptosis [13]. In the biomedical field, Ngb and Cygb have created considerable interest because these proteins appear to convey protection to cells and organs, e.g. after ischemia/reperfusion injury of the brain [14-16].

Due to the high number of available sequences, globins have become a popular model for the investigation of gene and gene family evolution [17]. In vertebrates, there are multiple *α-Hb *and *β-Hb *genes, which form distinct clusters. In birds and mammals, the *α-Hb *and *β-Hb *gene loci are found on separate chromosomes, while these loci are joined in fish and amphibians [18-20]. *Mb*, *Ngb *and *Cygb*, however, are typically single copy genes that are not associated with any other globin locus. Molecular phylogenetic studies and genomic comparisons may permit more refined insights into the function of Ngb and Cygb. Both of these proteins and genes are subject to strong purifying selection in all vertebrates studied so far, suggesting an essential cellular role [21,22]. Phylogenetic trees have shown that Ngb is distantly related to nerve hemoglobins in invertebrate worms, suggesting that its function is required in nerve cells of prototomian and deuterostomian animals, which diverged more than 600 million years ago [6]. Cygb, however, is a paralog of the muscle-specific Mb and may have been created by a duplication event only after the separation of agnathan and gnathostomian vertebrates about 450 million years ago [7]. In addition to the widespread Ngb and Cygb, some vertebrate lineages possess specific additional globin variants of unknown function, named globin × (GbX) in fish and frogs, globin Y (GbY) in frogs, lizards, and monotreme mammals and globin E (GbE) in birds [20,23-25]. To evaluate the resulting scenarios of vertebrate globin evolution, and to identify important, evolutionary conserved protein structure and ligand binding characteristics of human Ngb and Cygb, it is mandatory to identify and study candidate globin orthologs in non-vertebrate taxa.

In the 'new deuterostome phylogeny' [26,27], urochordates (tunicates) appear to be the closest relatives to vertebrates, while the cephalochordate amphioxus (lancelet), believed for a long time to be the vertebrate sister taxon, now appears to be basal to the vertebrate/tunicate clade. We have previously reported the globin gene repertoire of the tunicate *Ciona intestinalis *(sea squirt), consisting of at least four globins, clustered in a monophyletic clade. These genes are about equally distantly related to the vertebrate Ngb and GbX lineage, so that no clear orthology could be established [23,28]. In cephalochordates, the existence of a globin protein in notochord cells and myotome tissue of *Branchiostoma californiense *and *B. floridae *has been demonstrated by biochemical studies [29]. This intracellular globin is a dimer consisting of 19 kDa subunits with a high O_2 _affinity (P_50 _= 0.27 Torr, 15°C). Because of this high affinity and the absence of cooperativity, a possible role of the globins in facilitating diffusion of O_2 _into the notochord cells was discussed [29]. However, recent publications based on the genome sequence of *B. floridae *have yielded no hint at the presence of globin genes in this most basal chordate taxon [27,30]. To address this shortcoming, here we report the genomic organization of *B. floridae *globin genes (BflGb) and their evolutionary implications.

## Methods

### Database searches and sequence analyses

BLAST searches [31] were performed on whole genome shotgun data from the NCBI trace archive [32] and the *Branchiostoma floridae *genome project versions 1.0 and 2.0 at the JGI [33]. Searches of expressed sequence tags (ESTs) were performed using the *B. floridae *cDNA resource [34,35] and the NCBI EST database [36]. Nucleotide sequences were extracted from databases, assembled and translated using the DNAstar 5.08 program package (Lasergene).

Pairwise percentage sequence identities and similarities of proteins were calculated using the Matrix Global Alignment Tool (MatGAT) version 2.0 [37] using a PAM250 scoring matrix. Dotplots for detecting repeat structures were made using zPicture [38]. Prediction of subcellular localization of proteins was done by PSORT II [39]. N-myristoylation sites were predicted by Myristoylater [40].

### Molecular phylogeny

The conceptionally translated amino acid sequences of the *B. floridae *globins were manually added to an alignment of selected globin sequences [6,7]. The sequences used are *Homo sapiens *neuroglobin (HsaNGB [GenBank:AJ245946]), cytoglobin (HsaCYGB, [GenBank:AJ315162]), myoglobin (HsaMB [GenBank:M14603]), hemoglobin α (HsaHBA [GenBank:J00153]), and Hb β (HsaHBB [GenBank:M36640]); *Mus musculus *Ngb (MmuNgb [GenBank:AJ245946]), Cygb (MmuCygb [GenBank:AJ315163]) and Mb (MmuMb [GenBank:P04247]); *Ornithorhynchus anatinus *GbY (OanGbY [GenBank:AC203513]; *Gallus gallus *Ngb (GgaNgb [GenBank:AJ635192]), GbE (GgaGbE [GenBank:AJ812228]); *Taeniopygia guttata *GbE (TguGbE [XM_002196350]); *Xenopus tropicalis *GbX (XtrGbX [GenBank:AJ634915]), Hb α (XtrHbA [GenBank:P07428]), Hb β (XtrHbB [GenBank:P07429]), GbY (XtrGbY [GenBank:BC158411]); *Xenopus laevis *GbY (XlaGbY [GenBank:AJ635233]); *Danio rerio *Ngb (DreNgb [GenBank:AJ315610]), GbX (DreGbX [GenBank:AJ635194]), Cygb1 (DreCygb1 [GenBank:AJ320232]), Mb (DreMb [GenBank:AAR00323]); *Carassius auratus *GbX (CauGbX [GenBank:AJ635195]); *Myxine glutinosa *Hb1 (MglHb1 [GenBank:AF156936]), Hb3 (MglHb3 [GenBank:AF184239]); *Lampetra fluviatilis *Hb (LflHb [GenBank:P02207]); *Medicago sativa *leghemoglobin (MsaLegHb [GenBank:P09187]); *Lupinus luteus *leghemoglobin (LluLegHb [GenBank:P02240]); *Casuarina glauca *Hb1 (CglHb1 [GenBank:P08054]). In our deuterostome globin analysis, we refrained from inclusion of protostome globin sequences, because these tend to behave polyphyletically in the tree (Additional File 1), possibly due to long-branch attraction artifacts. Our trees were therefore rooted by plant globin sequences.

Phylogenetic tree reconstructions were performed using MrBayes version 3.1 [41,42] using the WAG model of amino acid evolution [43] assuming a gamma distribution of rates, as suggested by analysis of the alignment with ProtTest version 1.2.7 [44]. Metropolis-coupled Markov chain Monte Carlo sampling was performed with one cold and three heated chains that were run for up to 3,000,000 generations. Trees were sampled every 10^th ^generation and 'burn in' was set to 9,000. Maximum likelihood-based phylogenetic analysis was performed by RAxML version 7.2.3 [45] assuming the WAG model and gamma distribution of substitution rates. The resulting tree was tested by bootstrapping with 100 replicates.

### RT-PCR confirmation of *B. floridae *globin coding sequences

Adult specimens of *B. floridae *were collected at Tampa Bay, Florida, USA. Total RNA was isolated from whole animals using the RNeasy Kit according to the supplier's instructions (Qiagen). To remove genomic DNA a DNase I digestion step was included in the preparation. Reverse transcription of 0.5 μg total RNA was performed using SUPERSCRIPT II reverse transcriptase (Invitrogen) with an oligo(dT) primer. Using one-tenth of a cDNA reaction and 2 U Taq DNA polymerase (Sigma) the complete or partial coding sequences of the bioinformatically predicted globin genes were amplified in a standard PCR protocol. Missing 5' and 3' regions were obtained using the GeneRacer Kit with SUPERSCRIPT III reverse transcriptase (Invitrogen). PCR products were sequenced directly or were cloned into the pGEM-Teasy vector system (Promega) and both strands were sequenced by a commercial sequencing service (StarSeq). Nucleotide sequences were deposited in the database under the accession numbers listed in Table 1.

**Table 1:** Overview on amphioxus globin genes

globin	gene designation (Brafl genome V2.0)	alternative haplotype (Brafl genome version 1.0)	identity/similarity of haplotype proteins	EST data		RT-PCR confirmation of CDS	
				Acc.no. GenBank	expression	Acc.no. GenBank	coverage of CDS
**BflGb1**	BRAFLDRAFT_98913 [GeneID:7251500]	BRAFLDRAFT_92353	98.5/99.5%	-	-	FN773089	complete
**BflGb2**	BRAFLDRAFT_98914 [GeneID:7251501]	BRAFLDRAFT_92354	95.9/97.3%	-	-	FN773090	complete
**BflGb3**	BRAFLDRAFT_99970 [GeneID:7252875]	BRAFLDRAFT_72350	99.4/100%	-	-	FN773091	partial
**BflGb4**	BRAFLDRAFT_74626 [GeneID:7236365]	BRAFLDRAFT_84888	97.9/98.9%	-	-	FN773092	complete
**BflGb5**	BRAFLDRAFT_96953 [GeneID:7248969]	BRAFLDRAFT_110609	97.8/98.9%	BW852078	neurula whole animal	FN773093	complete
**BflGb6**	BRAFLDRAFT_74265 [GeneID:7230831]	BRAFLDRAFT_96513	100/100%	-	-	FN773094	partial
**BflGb7**	not annotated in database	not annotated in database	-	BW695259	adult whole animal	FN773095	complete
				BW701618	adult whole animal		
				BW702763	adult whole animal		
				BW704772	adult whole animal		
**BflGb8**	BRAFLDRAFT_92393 [GeneID:7215786]	BRAFLDRAFT_92377	97.6/99.4%			FN773096	partial
**BflGb9**	BRAFLDRAFT_96956 [GeneID:7250575]	not annotated in database	-	BW696096	adult whole animal	FN773097	complete
				BW700672	adult whole animal		
**BflGb10**	BRAFLDRAFT_118076 [GeneID:7214470]	BRAFLDRAFT_132915	97.2/98.6%	BW717063	adult whole animal	FN773098	complete
				BW695770	adult whole animal		
				BW698433	adult whole animal		
**BflGb11**	BRAFLDRAFT_127451 [GeneID:7247920]	BRAFLDRAFT_133418	98.6/99.3%	BW709730	adult whole animal	FN773099	complete
				BW700901	adult whole animal		
				BW706678	adult whole animal		
				BW695940	adult whole animal		
				BW720248	adult whole animal		
				BW721930	adult whole animal		
				BW709489	adult whole animal		
				BW717166	adult whole animal		
				BW693523	adult whole animal		
				BW692993	adult whole animal		
				BW710133	adult whole animal		
				BW699215	adult whole animal		
				BW698534	adult whole animal		
				BW709034	adult whole animal		
				BW701461	adult whole animal		
				BW703104	adult whole animal		
				AU234573	notochord (B. belcheri)		
**BflGb12**	BRAFLDRAFT_74222 [GeneID:7230826]	not annotated in database	-	-	-	FN773100	partial
**BflGb13**	BRAFLDRAFT_66937 [GeneID:7219951]	BRAFLDRAFT_111239	98.9/100%	-	-	FN773101	complete
**BflGb14**	BRAFLDRAFT_77082 [GeneID:7215245]	not annotated in database	-	-	-	FN773102	complete
**BflGb15**	BRAFLDRAFT_90360 [GeneID:7213766]	BRAFLDRAFT_90367	98.6/99.3%	-	-	FN773103	complete

Gene models, corresponding haplotypes, identities and similarities of haplotype proteins, nucleotide evolution rates of haplotypes, evidence for gene expression by ESTs and accession numbers of cDNA sequences are listed.

### Recombinant globin expression and characterization

Amphioxus globin variant BflGb4 coding sequence was isolated by RT-PCR, cloned into prokaryotic expression vector pET15b, verified by re-sequencing and ultimately expressed and affinity-purified from E. coli BL21pLys host cells by a Ni-NTA Superflow column (Qiagen). The kinetics of ligand binding by the flash photolysis method was measured to determine functional properties of the BflGb4 globin. After photolysis of the CO form, the subsequent completion of rebinding of the CO and any internal protein ligands provides information on the association and dissociation rates. Samples of 10 μM protein, on a heme basis, were placed under a controlled atmosphere of CO, oxygen or a mixture of both ligands, and photolyzed with 10 ns pulses at 532 nm.

## Results and Discussion

### Identification and annotation of *B. floridae *globin genes

**Figure 1:**
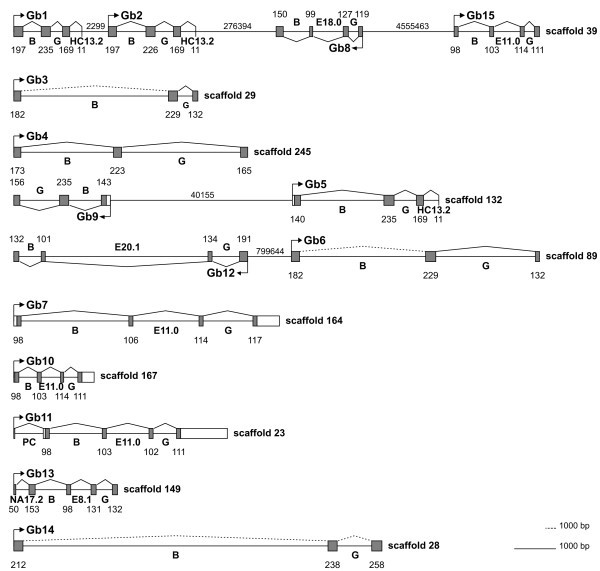
**Genomic organization of *B. floridae *globin (Gb) gene variants**. Only the haplotypes represented in genome assembly v.2.0 are shown and the genomic scaffolds are indicated. Exons are represented by grey boxes, connected by lines indicating mRNA splicing. Exon and intron sizes and distances between linked genes are indicated by numbers (in bp). Intron positions (bold letters) are given with reference to the globin helical structure (e.g. intron E8.1 is located between codon positions 1 and 2 of amino acid residue 8 in helix E).

Systematic TBlastN searches were carried out on the database of the *B. floridae *genome project versions 1.0 and 2.0 at the JGI [27] and the *Branchiostoma *cDNA resource [34] and complemented with the data of the whole genome shotgun sequences at the NCBI trace archive. Using vertebrate Ngb and Cygb sequences as query, fifteen distinct *bona fide *globin genes were identified in *Branchiostoma *genome v. 2.0, which reports a single haplotype at each locus [27] (Figure 1 Table 1). This is substantially more than the four gene copies identified in the tunicate *C. intestinalis *[28] but less than the largest globin gene families known from invertebrates (*C. elegans*: 33 genes [46]; *Chironomus *midges: 30-40 genes [47]). The reason for the higher globin gene copy number in *Branchiostoma *vs. *Ciona *is unknown. It may reflect differences in life-style (sand-burrowing vs. sessile) and/or the threefold higher genome size in amphioxus compared to the tunicate, which is thought to have undergone substantial gene loss [27]. Eight of the 15 gene models annotated in the database required extensive changes, which were performed by visual inspection of DNA sequencing traces, comparative amino acid sequence alignments and after cDNA verification by RT-PCR. One additional gene model (BRAFLDRAFT_81713) contains 86 amino acids of globin-related sequence embedded in a large protein of 1323 residues, annotated to be a calpain-like protease. We do not include this aberrant structure in the present analysis of *bona fide *globins.

Due to the still highly fragmented nature of the *Branchiostoma *genome draft, the picture of genomic organization of globin genes is currently incomplete. Only eight out of 15 gene copies co-localize to the same scaffold, with four globins being located on genomic scaffold 39 (*BflGb1*, *BflGb2*, *BflGb8 *and *BflGb15*). Here, *BflGb1 *and *BflGb2 *are situated in head-to-tail orientation only 2.3 kb apart from each other, and their amino acid similarity (83%; Additional file 2) may suggest their origin by a relatively recent gene duplication. The distance between *BflGb2 *and *BflGb8 *is 276 kb, between *BflGb8 *and *BflGb15 *even more than 4 Mbp, showing that amphioxus globin genes are widely disseminated instead of featuring the vertebrate-typical dense clustering. Genes *BflGb5 *and *BflGb9 *reside in head-to-head orientation separated by 40 kbp on scaffold 132 of the genome draft. This annotation is inconsistent with data of the trace archive, showing a head-to-tail orientation indicated by paired-end read information. *BflGb6 *and *BflGb12 *reside on scaffold 89 at a distance of 8 Mbp. All other seven globin genes are located on individual genomic scaffolds (Figure 1).

### Protein sequence comparisons and allelic differences

**Figure 2:**
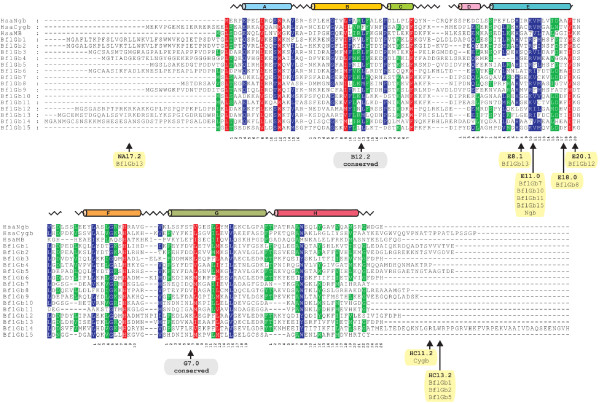
**Amino acid sequence alignment of inferred *B. floridae *globins (BflGb) and human (Hsa) globins Ngb, Cygb and Mb**. The globin α-helical structure is drawn on top of the alignment. Conserved positions are shaded. Intron positions in globin genes are indicated by arrows below the protein alignment and the intron presence in the respective gene variants is given.

The lengths of the deduced *Branchiostoma *globin sequences (Figure 2) range from 138 amino acids (BflGb11), which is consistent with the average length of the globin fold of 140-150 amino acids, to 236 residues (BflGb14). Such elongations, observed in 11 of the 15 amphioxus globins, result from N-and C-terminal extensions of the globin fold. The functional relevance of these extensions, previously reported e.g. for vertebrate Cygb [7] and nematode globins [46], is unclear. However, computer predictions using the PSORT II program [39] indicate that none of the amphioxus globins appears to contain a leader signal peptide, and all variants are predicted to be located in the cytoplasm. Notably, eight *Branchiostoma *globins (BflGb1, 2, 3, 6, 9, 12, 13, 14), five of which are phylogenetically related to vertebrate GbX (see below), possess a predicted N-myristoylation site. This may suggest an at least transient interaction with the cell membrane, thereby precluding an oxygen-supply function of these globins.

The comparison of the conceptionally translated amino acid sequences of *B. floridae *globins with human Ngb, Cygb and Mb shows the conservation of the typical functional residues of globins [1] in most of the amphioxus proteins, such as the distal histidine (amino acid position E7), the proximal histidine (F8) and the phenylalanine at CD1 (Figure 2). While the proximal histidine, which coordinates the heme iron atom, is conserved in all 15 putative globins, the distal histidine is replaced by leucine in BflGb6, 12 and 13. The same replacement was previously reported in globins of *Glycera dibranchiata *[48] and in nematodes [49,50]. It creates an unusually hydrophobic ligand-binding site and may reduce affinity for polar ligands like O_2 _[51]. The same *Branchiostoma *globin variants also show a change at amino acid position CD1 from phenylalanine to tryptophan, and BflGb3 displays an exchange of this residue by a tyrosine. Position CD1 (Phe) is even more conserved during globin evolution than the distal histidine. In human Hb, substitutions of CD1 (Phe) to non-aromatic amino acids usually lead to unstable globins and oxygenation problems [1]. The functional consequences of the more conservative CD1 changes in amphioxus variants, however, are unclear.

Pair-wise comparisons of the *Branchiostoma *globins display a substantial degree of divergence between the fifteen proteins. The most distant variants display a sequence identity of only 12% (BflGb11 and BflGb12) and a similarity of 31% (BflGb11 and BflGb14). As such, they are as distinct as vertebrate Mb and Cygb, which have separated before radiation of gnathostomes [21]. The most similar amphioxus paralogs (BflGb1 and BflGb2) have 60% sequence identity and 83% similarity (Additional file 2).

Possibly due to a large effective population size, *B. floridae *is highly heterozygous, and the genome sequencing of one specimen has revealed two haplotypes for two-thirds of the approximately 15,000 protein-coding loci [27]. We have identified allelic copies for 11 of the 15 globin variants (Table 1). Amino acid similarities/identities between alleles are high, ranging from 97/95% to 100%. Taking into account these interallelic comparisons, the overall conservation of the globins on the protein level and the expression evidence on the RNA level (see below), we propose that most, if not all 15 globin gene variants in amphioxus can be considered active genes and at least 12 genes may encode functional globin proteins.

### Evidence for globin gene expression

EST data provide evidence of transcription only for five *Branchiostoma *globin genes (*BflGb5*, *BflGb7*, *BflGb9*, *BflGb10 *and *BflGb11*; see Table 1). Represented by 17 EST entries, *BflGb11 *may be the most strongly expressed globin in whole adult animals. Based on the EST data and *in silico*-predictions, the coding regions of the fifteen genes were amplified by RT-PCR and completed by 5' and 3' RACE. Amplicons were cloned and sequenced for verification (Table 1). Together, these data demonstrate transcriptional expression of all *Branchiostoma *globin genes. Of special interest is the assignment of EST entry AU234573, representing *BflGb11*, to notochord tissue, as this facilitates further studies of the amphioxus globin components, which possibly serve to supply O_2 _to sustain the contractile function of notochord cells [29]. BflGb11, however, has a predicted molecular mass of 15 kDa and thus may not represent the major 19 kDa notochord globin fraction, as isolated biochemically by Bishop et al. [29]. Several other globin variants (e.g. BflGb5, 8, 9) have predicted molecular masses between 18 and 21 kDa.

### Identification of putative *Branchiostoma *orthologs to vertebrate globins

**Figure 3:**
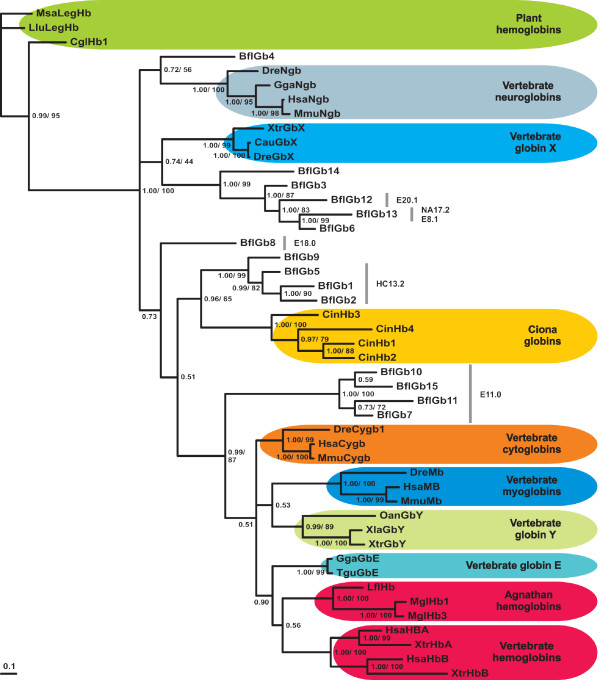
**Bayesian phylogenetic reconstruction reveals affiliations and possible orthologous relationships of *B. floridae *globin variants (bold face)**. Support values at branches are posterior probabilities and bootstrap percentages (> 40) of maximum likelihood analysis. Characteristic intron positions in the amphioxus globins are indicated and in some cases confirm monophyletic clades of BflGb paralogs. Species abbreviations are listed in the Material and Methods section.

**Figure 4:**
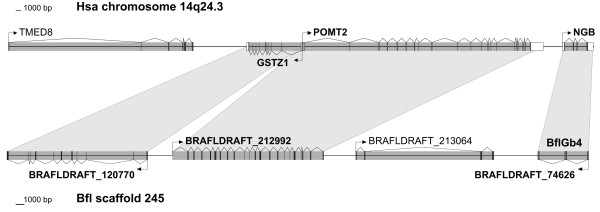
**Conserved syntenic relationships of the *B. floridae *genomic region encompassing the *BflGb4 *gene and human (Hsa) chromosome 14, containing the *NGB *gene**.

The amino acid sequences of the 15 globin genes of *Branchiostoma *were included in an alignment of selected vertebrate and invertebrate globins [6,7,28]. Bayesian and maximum likelihood phylogenetic reconstruction revealed possible orthology relationships between amphioxus and vertebrate globins (Figure 3). Most importantly, BflGb4 forms a common clade with vertebrate Ngb. These two globins show 27%/49% identity/similarity. Corroborating evidence for orthology was obtained by inspecting the organization at the genomic level. Within vertebrates, the gene region containing *Ngb *is strongly conserved in gene order and arrangement [20,22]. The human *NGB *gene resides on chromosome HSA14q24.3 between the genes for protein-O-mannosyltransferase 2 (*POMT2*) and transmembrane protein 63C (*TMEM63C*, previously termed *DKFZp434P0111*) [22]. While a *TMEM63C *ortholog was not detectable on genomic scaffold 245 containing *BflGb4*, the amphioxus orthologs of *POMT2 *and *glutathione transferase zeta 1 *(*GSTZ1*), located on the distal, telomeric side of the human *NGB *gene, reside in close proximity to the *BflGb4 *gene (Figure 4). These findings are in agreement with Putnam et al. [27], who reported extensive micro-syntenic conservation of gene arrangement between amphioxus and humans on the whole-genome level. Together with the phylogeny, the data are convincing evidence that we have identified an ortholog of vertebrate Ngb in a basal chordate.

Phylogenetical interpretation of the tree reconstruction (Figure 3) further suggests that the monophyletic clade comprising BflGb3, BflGb6, BflGb12, BflGb13 and BflGb14 contains the putative orthologs of vertebrate GbX, a distant relative of the Ngb lineage, which is found only in fish and frogs, but not in birds and mammals [20,23]. This corroborates the scenario that GbX was already present in early chordates, but has been lost secondarily during tetrapod evolution. Syntenic conservation of *GbX *flanking genes in *Xenopus tropicalis *and *Tetraodon nigroviridis *is restricted to three proximate genes encoding a pleckstrin domain containing protein (PLEKHG), phospholipase and signal recognition particle SRP12 [20]. Notably the genome of *B. floridae *revealed the linkage of a *PLEKHG *gene to *BflGb3*, substantiating the assumption of a possible *GbX *orthology (Additional file 3).

Another amphioxus globin clade comprising BflGb1, BflGb2, BflGb5 and BflGb9 is paralogous to all other vertebrate globins (Hb, Mb, Cygb). This clade also groups with the four monophyletic globin variants from the genome of the tunicate *C. intestinalis *[28], which confirms our view that the *C. intestinalis *globins are not 1:1 orthologous to vertebrate globins.

A third monophyletic group of amphioxus globin variants, comprising BflGb7, BflGb10, BflGb11 and BflGb15, joins the vertebrate Mb-Hb-Cygb-GbE-GbY lineage in the tree. It is noteworthy that this clade of amphioxus globins contains BflGb11, which may be expressed in notochord tissue, possibly serving the Mb-like role in O_2 _supply suggested by Bishop et al. [29]. Unfortunately, analysis of syntenic gene relationships of the *Mb*, *Cygb *and *Hb *loci [19,22,52] did not generate further positive evidence for 1:1 gene orthology between amphioxus and vertebrate globins, possibly due to the fragmentary nature of the draft genome assembly. The absence of clear *Cygb*, *Mb*, *Hb *and GbE/Y orthologues in *Branchiostoma *may confirm that the gene duplications, which gave rise to these diverse vertebrate globin types, indeed happened after the split of cephalochordates and the vertebrate ancestor [7]. Nevertheless, the phylogenetic predictions will facilitate guided functional comparisons of cephalochordate and vertebrate globins.

### Implications for the evolution of the ancestral vertebrate globin gene cluster

According to the current model of vertebrate globin evolution, the mammalian *α-Hbs *constitute the ancestral vertebrate globin gene locus, while the β-cluster is the result of a transposition of globin genes into a region containing olfactory receptor (OR) genes [24,52]. Looking deeper into the evolutionary past, Wetten et al. [53] suggested a common origin of the vertebrate α-*Hb *locus and two globin gene-containing regions in the *C. intestinalis *genome, as evidenced by the syntenic relationships of three globin-flanking genes. However, these genes do not show linkage to globins in the amphioxus genome. Wetten et al. [53] therefore proposed that they were secondarily linked to globin genes by a fusion of conserved genomic linkage groups (CLGs 3, 15 and 17) [27] to produce the ancestor of the vertebrate *α-Hb *locus before the divergence of urochordates and vertebrates. Our own analyses of gene synteny, however, do not provide support for this model, since we could not detect any of the *B. floridae *globin genes within the respective CLGs. Instead, we find that *Branchiostoma *globins *BflGb5 *and *9 *reside on the same genomic scaffold as the amphioxus orthologue of *integrin-linked kinase *(*ILK*), a conserved flanking gene of the *β-Hb *cluster in men, chicken and marsupials [24].

Additionally, a detailed inspection of the CLG's architecture [27] reveals that the amphioxus genomic scaffold including *BflGb1*, *2*, *8 *and *15 *corresponds to human chromosomal region 11p15.4-15.5, the location of the *β-Hb *cluster in man. Of course, this hypothetical ancient orthology is at odds with the transpositional model for a more recent origin of the *β-Hb *locus during vertebrate evolution (the olfactory receptor genes are dispersed in amphioxus [54], and thus cannot help to clarify the evolutionary events). Clearly, a reliable reconstruction of the pre-vertebrate globin loci will require the analysis of additional deuterostome genomes.

### Kinetics of ligand binding of the putative Ngb ortholog, BflGb4

**Figure 5:**
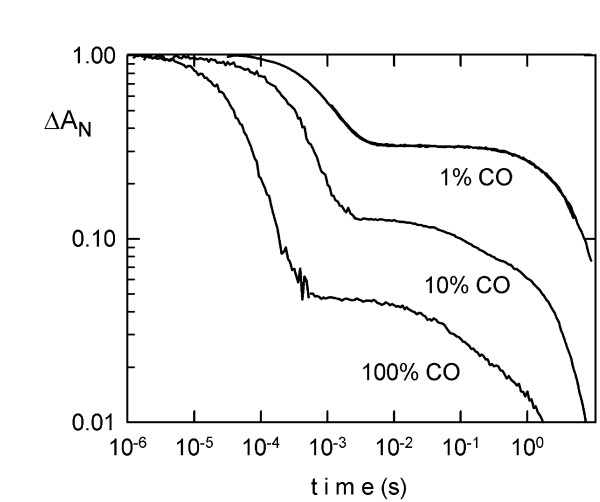
**Ligand binding kinetics of recombinantly expressed BflGb4**. After photodissociation of CO, the kinetics of ligand rebinding are biphasic, as previously observed for other hexacoordinated globins such as vertebrate Ngb. During the rapid μs phase, CO and the internal ligand (His) compete for the binding to the transient pentacoordinated heme. For the fraction that binds His, there is a slow replacement of His to CO to return to the original stable form. Measurements at different CO concentrations (and several detection wavelengths-not shown) allow a determination of the association rate for both ligands and the dissociation rate of His. The deltaA_N _on the Y-axis represents the normalized change in absorbance.

The ligand binding kinetics of recombinantly expressed BflGb4 after CO photodissociation is biphasic (Figure 5), as previously observed for Ngb and Cygb [55,56]. The rapid phase is the competitive binding of CO and the internal ligand (considered to be the distal E7 histidine of the globin fold), and can be simulated by a bimolecular reaction with CO and a fixed rate of 4000/s for the protein ligand. From the slow phase, a rate for histidine dissociation of 2/s was extracted. This indicates that BflGb4 globin, like its vertebrate ortholog Ngb [55,57], is a hexacoordinated globin, which adopts a His-Fe-His coordination in the absence of external ligands. The hexacoordination of the heme Fe atom in BflGb4 underlines the view that this binding scheme represents an ancestral feature of animal and plant globins, from which penta-coordinated globins like Hb and Mb evolved [58]. The exact adaptive value of hexacoordination is still unclear. However, it may confer some unusual thermal and acidosis stability to the globin fold [59,60], which could be relevant under environmental or cellular stress.

The CO and O_2 _ligand association rates of BflGb4 are quite high (as for Ngb, but not Cygb; [61]), which places the amphioxus globin with Ngb in terms of ligand binding kinetics. The overall oxygen affinity of BflGb4 (oxygen half-saturation value P_50 _of 3 Torr at 25°C) is about twice that for human NGB (without the disulfide bond [61]), due to a higher intrinsic O_2 _affinity. However, values are close enough to suggest similar functional roles of the orthologous proteins. In contrast to human NGB, BflGb4 apparently lacks internal cysteine residues, which have been hypothesized to modulate oxygen affinity depending on the cellular redox state [61]. Note that the oxygen affinity for BflGb4 is intermediate to the two allosteric states of human NGB, and there is preliminary evidence in the kinetics of BflGb4 of an additional conformational state. Clearly, further detailed comparisons are needed to extract conserved and taxon-specific features of these globins.

### Globin intron evolution and the presence of minisatellites in amphioxus globin genes

The ancestry and conservation of globins has stimulated studies to trace the evolutionary behavior of introns in these genes, aiming at contributing to the long-standing introns-early versus introns-late debate [18,62-64]. Two introns at positions B12.2 (i. e. between codon positions 2 and 3 of the 12^th ^amino acid of globin helix B) and G7.0 are conserved in all vertebrate globins, in many invertebrate and even plant globin genes. They are therefore thought to have already existed in the globin gene ancestor [65]. Both these intron positions can also be found conserved in all 15 amphioxus globin genes (Figure 1, 2). In addition to the strictly conserved B12.2 and G7.0 introns, there are introns at slightly differing positions of the globin E-helix ("central introns") present in globin genes of diverse taxa (vertebrates, invertebrates and plants), which raised speculations on the presence of such a central intron already in the globin ancestor [18,62]. Subsequent findings of different E-helix introns in globin genes of closely related insect species casted doubt on this view and argued for an intron gain scenario [64]. Interestingly, the amphioxus globin genes reveal four different intron positions within the E-helix (E8.1, E11.0, E18.0, E20.1; Figure 1 and 2), of which only positions E11.0 (in vertebrate *Ngb*; [6]) and E18.0 (in nematode globins; [46,66]) have been reported before. This situation can in principle be explained by a positional shift of ancestral introns (= "intron sliding"), intron loss or insertional intron gain. Intron sliding, however, is thought to explain only very small intron shifts [23,67]. An intron loss scenario would require many such independent events on several branches of the phylogenetic tree (Figure 3). Therefore, the most parsimonious explanation is that the divergent central intron positions in globin genes in amphioxus and other taxa are due to convergent intron gain. This is corroborated by the lack of a central intron in *BflGb4*, the amphioxus ortholog of vertebrate *Ngb*. Intron gain may also be responsible for the presence of introns at the unprecedented positions HC13.2 (between H-helix and C-terminus) in amphioxus globin gene variants *BflGb1*, *2 *and *5 *and intron position NA17.2 (between N-terminus and A helix) in gene *BflGb13 *(Figure 1).

**Figure 6:**
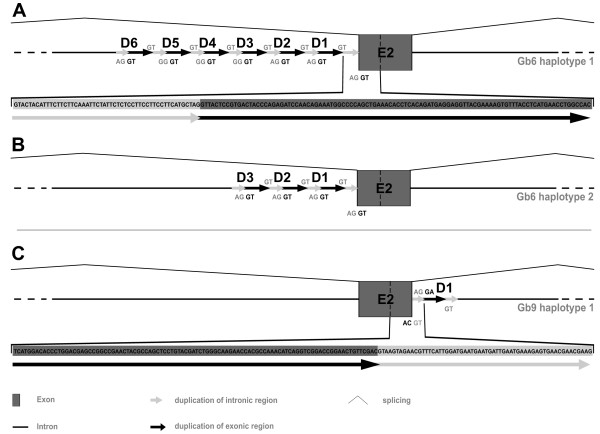
**Minisatellite-like tandem duplications of intron/exon boundaries (termed 'mirages') in amphioxus globin genes *BflGb6 *(A, haplotype 1; B, haplotype 2) and BflGb9 (C)**. Note that both, the 5' and the 3' boundaries can undergo such duplication events.

Detailed annotation of the genomic organization of amphioxus globin genes revealed conspicuous structures, which are interesting with respect to intron evolution. In gene *BflGb6 *we observed minisatellite-like tandem duplications, comprising the 3' end of the B12.2 intron and the 5' part of exon 2, while *BflGb9 *contains a duplicate of the 3' boundary of exon 2 (Figure 6). Such tandem repeats spanning an exon-intron boundary have previously been reported in the *alcohol dehydrogenase 3 *(*Adh3*) gene of *B. floridae *and *B. lanceolatum *and have been termed "mirages" [68]. Other gene loci with similar structures have been reported in amphioxus [69,70], possibly making this a more general phenomenon in cephalochordates. The genomic mechanism of generation of these minisatellites is unclear, and repeat units vary in length (*BflGb6*: 150-160 bp, *BflGb9*: 157 bp; *Adh3*: 10-72 bp, [67]). The *Adh3 *data as well as our globin RT-PCR results suggest that mirage structures do not interfere with regular splicing of the mRNA, although many, but not all of the tandem repeats contain AG/GT splice signals (for the *BflGb6 *example, see Figure 6 and Additional file 4) which could be used as cryptic splice sites producing alternative (and possibly aberrant) transcripts. Like other minisatellites, mirage clusters display length instability, possibly due to unequal or intra-strand crossing-overs, and even somatic instability has been detected at the *Adh3 *locus [68]. For haplotypes 1 and 2 of *BflGb6*, we have observed 6 and 3 repeat units, respectively (Figure 6; no second haplotype was found for *BflGb9*). The repeat units of *BflGb6 *display between 81 and 100% nucleotide sequence identity (Additional file 5). Reconstruction of cluster evolution by phylogenetic trees (Additional file 6) reveals typical features of tandem repeat turnover [71], namely concerted evolution within clusters (units D1/D2 of haplotype 2 and D3/D4/D5 of haplotype 1) and exclusion of cluster boundaries (D6/haplotype 1 and D3/haplotype 2) from such intra-allelic homogenization.

**Figure 7:**
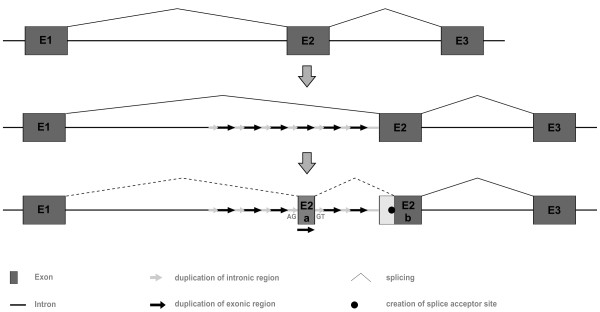
**Hypothetical model for the creation of a novel globin intron from intragenic mirage duplications**. A mirage repeat from the central part of the cluster is recruited to form exon E2a and be spliced to exon E2b, if a suitable splice acceptor site (black dot) is present. The novel splicing events are shown by dashed lines. The model predicts that newly generated introns contain mirage-derived repeats, which however may degenerate by mutation.

With respect to the evolution of introns, mirage repeats immediately offer a suggestive model to explain intron gain within the globin genes over evolutionary times (Figure 7). An exonic part of a repeat unit (e.g. D3) may secondarily turn into a real exon, if its boundary cryptic splice signals are being used. If a suitably positioned splice acceptor site is already present or created by mutation within the original exon 2, the mirage repeats in between will become intronic. In support of this model, we recognize degenerate tandem repeats within the hypothetically gained intron E8.1 of *BflGb13 *(Additional file 7). The general idea of intron gain by duplication events encompassing AG/GT proto-splice sites was originally introduced by Rogers [72] and has received renewed interest by studies of intron evolution in ray-finned fishes [73] and mammals [74]. Recently, the systematic examination of six fully sequenced model organism genomes including humans, mouse and *Drosophila *has emphasized the importance of internal gene duplications as a mechanism for intron generation [75].

## Conclusions

The identification of putative orthologs of vertebrate globin variants Ngb, GbX and the Mb/Cygb/Hb lineage in the *B. floridae *genome emphasizes the particular value of cephalochordates as a reference taxon for vertebrate evolution. Although the urochordate lineage may be overall more closely related to vertebrates, the tunicate (*C. intestinalis*) does not appear to harbour any 1:1 orthologs of vertebrate globin genes [28]. The present study facilitates detailed functional studies of the amphioxus globins in order to trace conserved properties and specific adaptations of respiratory proteins at the base of chordate evolution.

## Authors' contributions

BE carried out the bioinformatic and molecular genetic analyses, and drafted the manuscript. GP performed RT-PCR on amphioxus specimen. LK and MCM performed ligand-binding studies and co-wrote the paper. SV and TB contributed to the initial bioinformatic work of gene identification. TH conceived of the study, and wrote the final version of the manuscript. All authors read and approved this final manuscript.

## Supplementary Material

Additional file 1 **Bayesian phylogenetic reconstruction based on globin alignment including protostome sequences**. *Lumbricus terrestris *globin B (LteGbB [GenBank:P02218]) and globin D (LteGbD [GenBank:P08924), *Glycera dibranchiata *hemoglobin P3 (GdiHbP3; P23216), *Drosophila melanogaster *globin 1 (DmeGlob1 [GenBank:AJ132818]), *Chironomus thummi *hemoglobin VI (CttHbVI [GenBank:P02224]) show paraphyletic distribution and therefore were excluded from the analysis shown in Figure 3. Posterior probabilities are indicated at branches. Click here for file

Additional file 2 **Amino acid similarities (lower half) and identities (upper half) of amphioxus globin variants and selected vertebrate globins**. Click here for file

Additional file 3 **Conserved syntenic relationships of the *B. floridae *genomic region encompassing the *BflGb3 *gene and human (Hsa) chromosome 14, containing the *NGB *gene**. Click here for file

Additional file 4 **Nucleotide sequence alignment of the minisatellite-like exon/intron boundary duplications (termed 'mirages') of globin gene *BflGb6 *haplotype 1 (upper part) and hyplotype 2 (lower part)**. The light grey line above the alignment shows the intronic part with the splice acceptor site, the darker grey line shows the exonic part of the duplicated structures. Duplicates are designated D1-D6, while the authentic genic sections, which form part of the gene transcripts, are named "E2+intron". Note that the *BflGb6 *mirage structure from haplotype 1 was reconstructed by us using trace sequence data, while the version within the genome draft appears to be aberrantly assembled. Click here for file

Additional file 5 **Nucleotide sequence identity of mirage repeats from *BflGb6***. For designations, see Additional file 4. Click here for file

Additional file 6 **Reconstruction of repeat relationships by a neighbor-joining phylogenetic tree**. For designations, see Additional file 4. Click here for file

Additional file 7 **Dot plot nucleotide sequence comparison of the *BflGb13 *gene region comprising exons 3 and 4 (E3, E4) and the encompassed 'central' E8.1 intron (with itself)**. Both haplotypes show degenerate repeats in the intron, which might indicate intron origin from mirage-type duplications. Click here for file
