# Supplementary figures and images for: The globin gene family of the cephalochordate amphioxus: implications for chordate globin evolution
Source: BMC Evol Biol. 2010 Nov 30;10:370. doi: 10.1186/1471-2148-10-370 (PMC3087553; doi:10.1186/1471-2148-10-370)

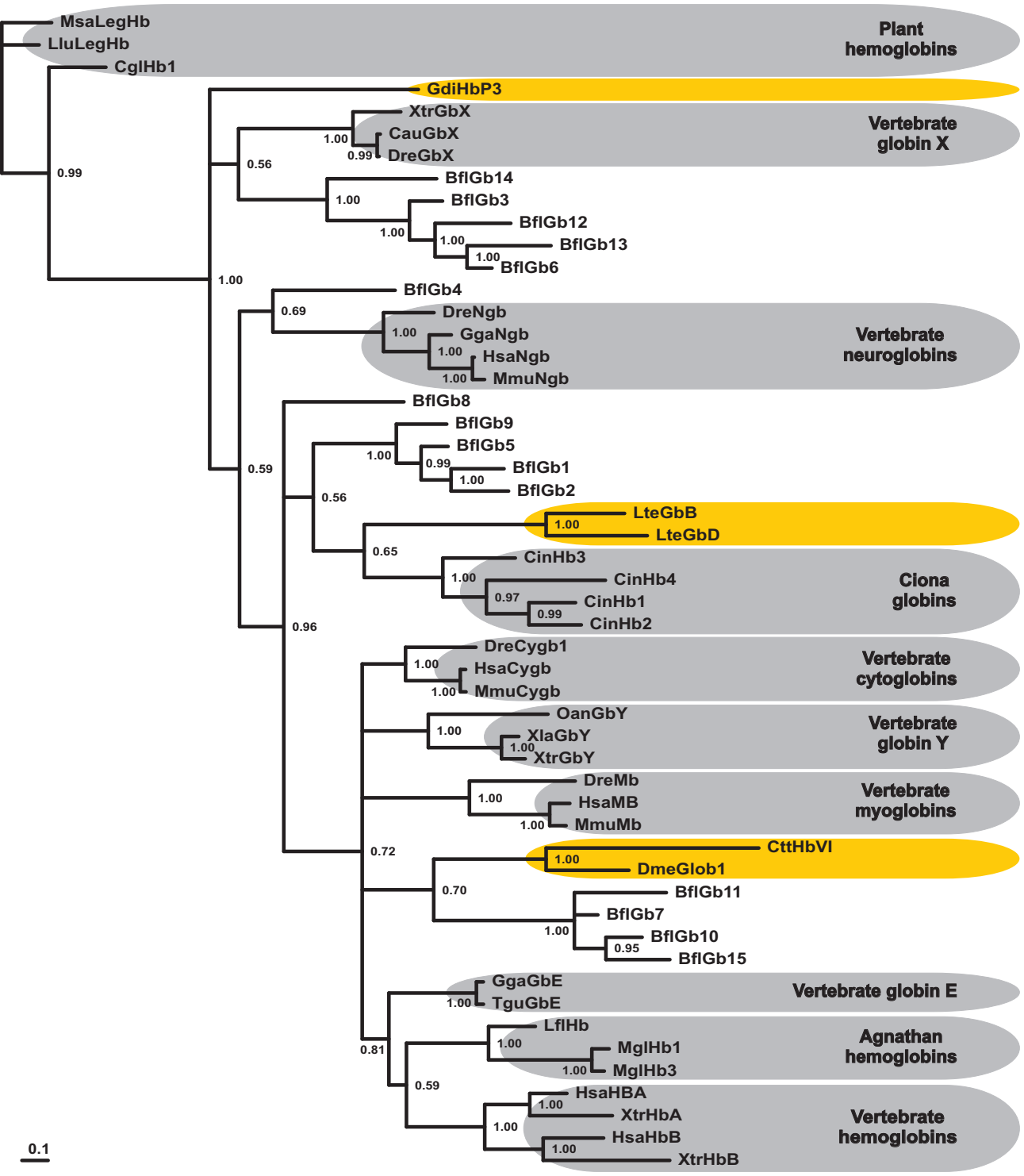

Supplement: Additional file 1 — Bayesian phylogenetic reconstruction based on globin alignment including protostome sequences. Lumbricus terrestris globin B (LteGbB [GenBank:P02218]) and globin D (LteGbD [GenBank:P08924), Glycera dibranchiata hemoglobin P3 (GdiHbP3; P23216), Drosophila melanogaster globin 1 (DmeGlob1 [GenBank:AJ132818]), Chironomus thummi hemoglobin VI (CttHbVI [GenBank:P02224]) show paraphyletic distribution and therefore were excluded from the analysis shown in Figure 3. Posterior probabilities are indicated at branches. [file 1471-2148-10-370-S1.PDF]

1000 bp **Xtr scaffold 37**

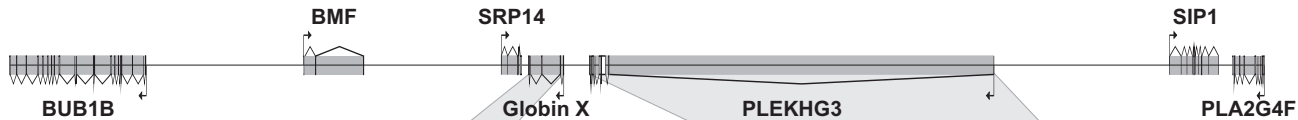

1000 bp **Bfl scaffold 29**

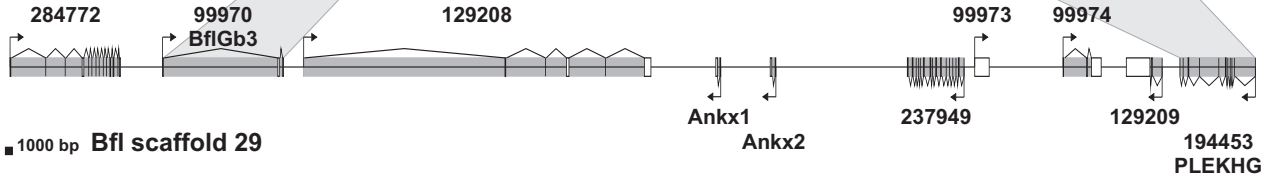

Supplement: Additional file 3 — Conserved syntenic relationships of the B. floridae genomic region encompassing the BflGb3 gene and human (Hsa) chromosome 14, containing the NGB gene. [file 1471-2148-10-370-S3.PDF]

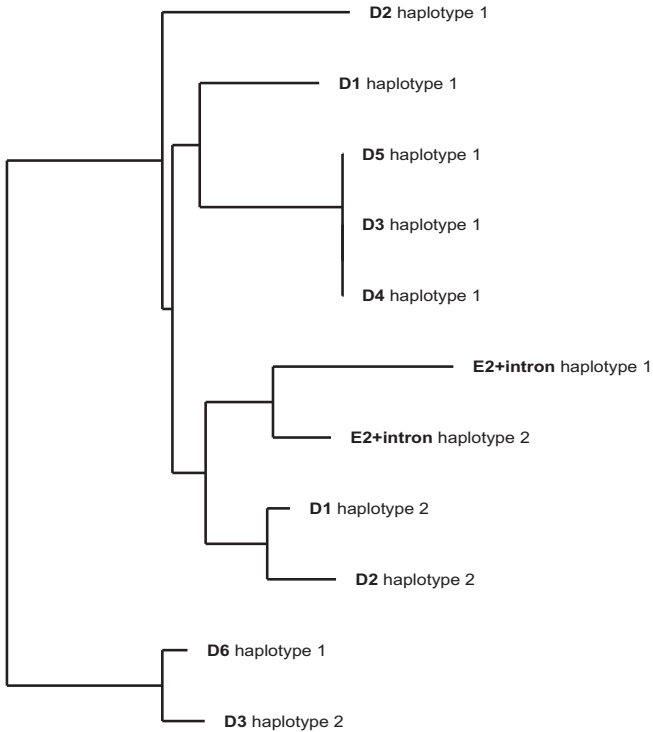

Supplement: Additional file 6 — Reconstruction of repeat relationships by a neighbor-joining phylogenetic tree. For designations, see Additional file 4. [file 1471-2148-10-370-S6.PDF]

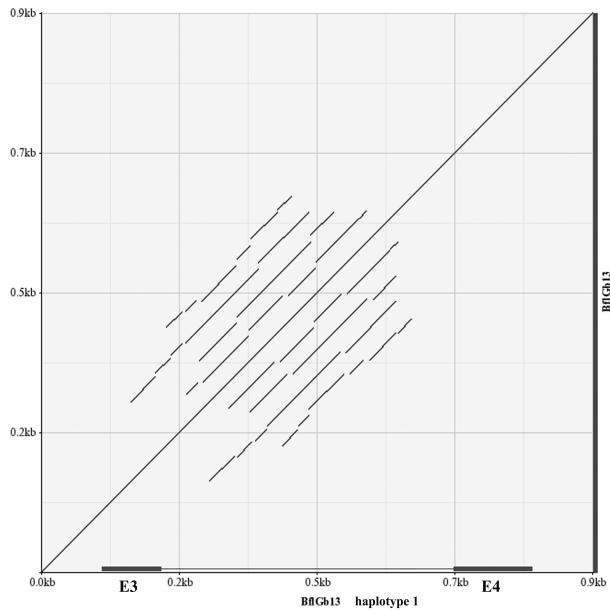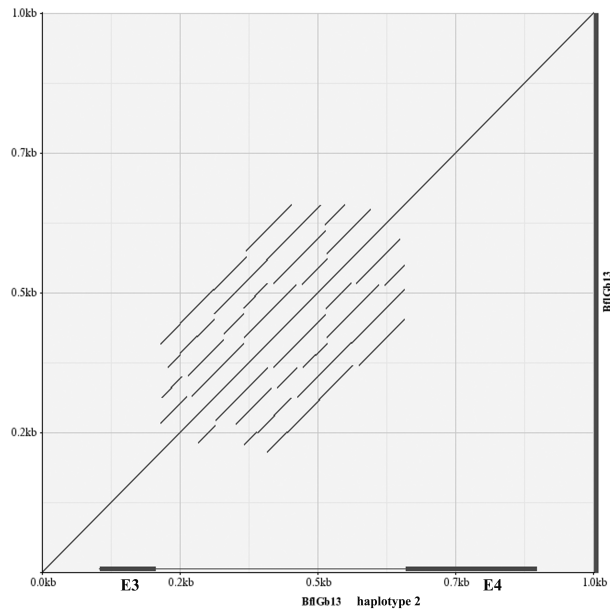

Supplement: Additional file 7 — Dot plot nucleotide sequence comparison of the BflGb13 gene region comprising exons 3 and 4 (E3, E4) and the encompassed 'central' E8.1 intron (with itself). Both haplotypes show degenerate repeats in the intron, which might indicate intron origin from mirage-type duplications. [file 1471-2148-10-370-S7.PDF]
