# Supplementary material for: The globin gene family of the cephalochordate amphioxus: implications for chordate globin evolution
Source: BMC Evol Biol. 2010 Nov 30;10:370. doi: 10.1186/1471-2148-10-370 (PMC3087553; doi:10.1186/1471-2148-10-370)
Supplement: Additional file 4 — Nucleotide sequence alignment of the minisatellite-like exon/intron boundary duplications (termed 'mirages') of globin gene BflGb6 haplotype 1 (upper part) and hyplotype 2 (lower part). The light grey line above the alignment shows the intronic part with the splice acceptor site, the darker grey line shows the exonic part of the duplicated structures. Duplicates are designated D1-D6, while the authentic genic sections, which form part of the gene transcripts, are named "E2+intron". Note that the BflGb6 mirage structure from haplotype 1 was reconstructed by us using trace sequence data, while the version within the genome draft appears to be aberrantly assembled. [file 1471-2148-10-370-S4.PDF]

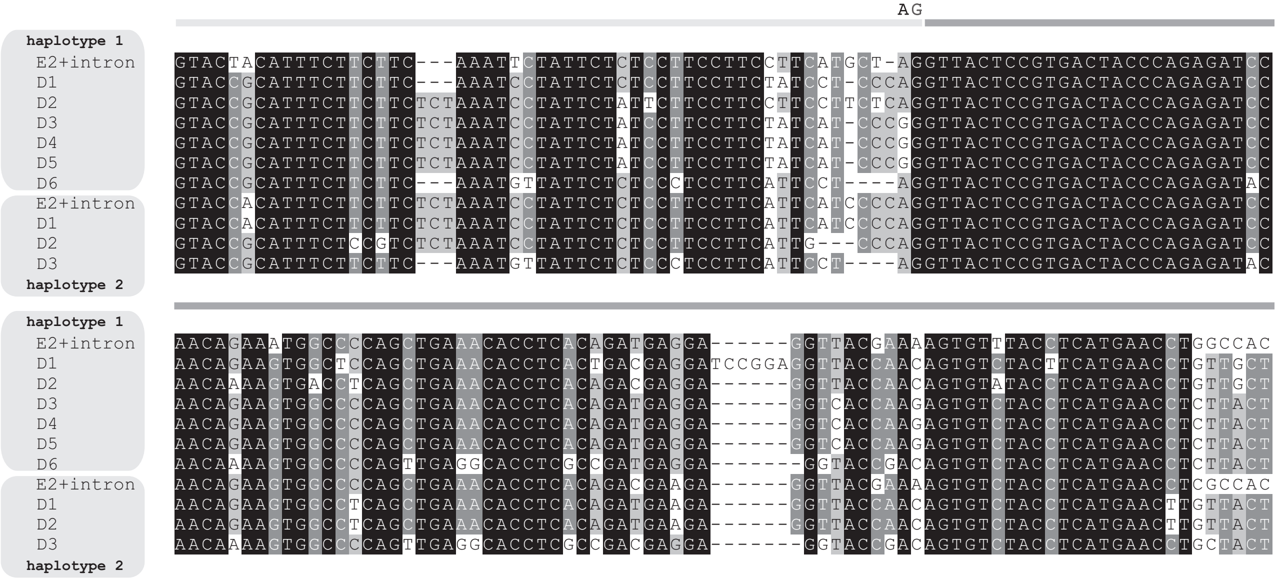

AG
haplotype 1
E2+intron GTACTACATTTCTTCTTC---AAATTCTATTCTCTCCTTCCTTCCTTCATGCT-AGGTTACTCCGTGACTACCCAGAGATCC
D1 GTACCGCATTTCTTCTTC---AAATCCTATTCTCTCCTTCCTTCTATCCT-CCCAGGTTACTCCGTGACTACCCAGAGATCC
D2 GTACCGCATTTCTTCTTCTCTAAATCCTATTCTATTCTTCCTTCCTTCCTTCTCAGGTTACTCCGTGACTACCCAGAGATCC
D3 GTACCGCATTTCTTCTTCTCTAAATCCTATTCTATCCTTCCTTCTATCAT-CCCGGGTTACTCCGTGACTACCCAGAGATCC
D4 GTACCGCATTTCTTCTTCTCTAAATCCTATTCTATCCTTCCTTCTATCAT-CCCGGGTTACTCCGTGACTACCCAGAGATCC
D5 GTACCGCATTTCTTCTTCTCTAAATCCTATTCTATCCTTCCTTCTATCAT-CCCGGGTTACTCCGTGACTACCCAGAGATCC
D6 GTACCGCATTTCTTCTTC---AAATGTTATTCTCTCCCTCCTTCATTCCT----AGGTTACTCCGTGACTACCCAGAGATAC
E2+intron GTACCACATTTCTTCTTCTCTAAATCCTATTCTCTCCTTCCTTCATTCATCCCCAGGTTACTCCGTGACTACCCAGAGATCC
D1 GTACCACATTTCTTCTTCTCTAAATCCTATTCTCTCCTTCCTTCATTCATCCCCAGGTTACTCCGTGACTACCCAGAGATCC
D2 GTACCGCATTTCTCCGTCTCTAAATCCTATTCTCTCCTTCCTTCATTG---CCCAGGTTACTCCGTGACTACCCAGAGATCC
D3 GTACCGCATTTCTTCTTC---AAATGTTATTCTCTCCCTCCTTCATTCCT----AGGTTACTCCGTGACTACCCAGAGATAC
haplotype 2
haplotype 1
E2+intron AACAGAAATGGCCCCAGCTGAAACACCTCACAGATGAGGA------GGTTACGAAAGTGTTTACCTCATGAACCTGGCCAC
D1 AACAGAAGTGGCTCCAGCTGAAACACCTCACTGACGAGGATCCGGAGGTTACCAACAGTGTCTACTTCATGAACCTGTTGCT
D2 AACAAAAGTGACCTCAGCTGAAACACCTCACAGACGAGGA------GGTTACCAACAGTGTATACCTCATGAACCTGTTGCT
D3 AACAGAAGTGGCCCCAGCTGAAACACCTCACAGATGAGGA------GGTCACCAAGAGTGTCTACCTCATGAACCTCTTACT
D4 AACAGAAGTGGCCCCAGCTGAAACACCTCACAGATGAGGA------GGTCACCAAGAGTGTCTACCTCATGAACCTCTTACT
D5 AACAGAAGTGGCCCCAGCTGAAACACCTCACAGATGAGGA------GGTCACCAAGAGTGTCTACCTCATGAACCTCTTACT
D6 AACAAAAGTGGCCCCAGTTGAGGCACCTCGCCGATGAGGA-------GGTACCGACAGTGTCTACCTCATGAACCTCTTACT
E2+intron AACAGAAGTGGCCCCAGCTGAAACACCTCACAGACGAAGA------GGTTACGAAAGTGTCTACCTCATGAACCTCGCCAC
D1 AACAGAAGTGGCCTCAGCTGAAACACCTCACAGATGAAGA------GGTTACCAACAGTGTCTACCTCATGAACTTGTTACT
D2 AACAGAAGTGGCCTCAGCTGAAACACCTCACAGATGAAGA------GGTTACCAACAGTGTCTACCTCATGAACTTGTTACT
D3 AACAAAAGTGGCCCCAGTTGAGGCACCTCGCCGACGAGGA-------GGTACCGACAGTGTCTACCTCATGAACCTGCTACT
haplotype 2
